# *catena*-Poly[[[bis­[2,2′-(propane-1,3-diyl­dithio)bis­(1,3,4-thia­diazole)-κ*N* ^4^]copper(II)]-bis­[μ-2,2′-(propane-1,3-diyldithio)bis­(1,3,4-thia­diazole)-κ^2^ *N* ^4^:*N* ^4′^]] bis­(perchlorate)]

**DOI:** 10.1107/S1600536809006722

**Published:** 2009-02-28

**Authors:** Jian-Hua Qin, Jian-Ge Wang, Pu-Zhou Hu

**Affiliations:** a College of Chemistry and Chemical Engineering, Luoyang Normal University, Luoyang 471022, People’s Republic of China

## Abstract

In the title compound, {[Cu(C_7_H_8_N_4_S_4_)_4_](ClO_4_)_2_}_*n*_, the Cu^II^ atom, occupying a crystallographic inversion centre, is six-coordinated by six N atoms of three symmetry-related 2,2′-(propane-1,3-diyldithio)bis­(1,3,4-thia­diazole) (*L*) ligands in a slightly distorted octa­hedral geometry. The ligand *L* adopts two kinds of coordination modes in the crystal structure; one is a monodentate coordination mode and serves to complete the octa­hedral coordination of the Cu atom and the other is an *N*:*N*′-bidentate bridging mode in a *trans* configuration, bridging Cu atoms *via* translation symmetry along the *b* axis into a chain structure. The perchlorate ions serve as acceptors for inter­molecular C—H⋯O hydrogen bonds, which link the chains into a three-dimensional network.

## Related literature

For Cu—N bonds see, for example: Huang *et al.* (2009 ▶); Wang *et al.* (2008 ▶). 
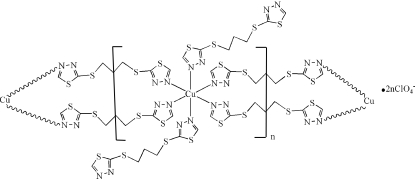


## Experimental

### 

#### Crystal data

[Cu(C_7_H_8_N_4_S_4_)_4_](ClO_4_)_2_
*M* *_r_* = 1368.10
Triclinic, 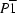

*a* = 10.321 (3) Å
*b* = 11.122 (3) Å
*c* = 12.908 (4) Å
α = 67.213 (3)°
β = 76.602 (3)°
γ = 76.675 (3)°
*V* = 1312.3 (6) Å^3^
*Z* = 1
Mo *K*α radiation
μ = 1.22 mm^−1^
*T* = 294 K
0.39 × 0.28 × 0.24 mm

#### Data collection

Bruker SMART CCD area-detector diffractometer
Absorption correction: multi-scan (*SADABS*; Bruker, 1997 ▶) *T*_min_ = 0.646, *T*_max_ = 0.756
9833 measured reflections
4857 independent reflections
4081 reflections with *I* > 2σ(*I*)
*R*_int_ = 0.017

#### Refinement

*R*[*F* ^2^ > 2σ(*F* ^2^)] = 0.040
*wR*(*F* ^2^) = 0.110
*S* = 1.03
4857 reflections
322 parameters
H-atom parameters constrained
Δρ_max_ = 1.05 e Å^−3^
Δρ_min_ = −0.50 e Å^−3^

### 

Data collection: *SMART* (Bruker, 1997 ▶); cell refinement: *SAINT* (Bruker, 1997 ▶); data reduction: *SAINT*; program(s) used to solve structure: *SHELXS97* (Sheldrick, 2008 ▶); program(s) used to refine structure: *SHELXL97* (Sheldrick, 2008 ▶); molecular graphics: *SHELXTL* (Sheldrick, 2008 ▶); software used to prepare material for publication: *SHELXTL*.

## Supplementary Material

Crystal structure: contains datablocks I, global. DOI: 10.1107/S1600536809006722/si2158sup1.cif

Structure factors: contains datablocks I. DOI: 10.1107/S1600536809006722/si2158Isup2.hkl

Additional supplementary materials:  crystallographic information; 3D view; checkCIF report

## Figures and Tables

**Table 1:** Selected bond lengths (Å)

Cu1—N1	2.021 (2)
Cu1—N5	2.053 (2)
Cu1—N4^i^	2.445 (3)

Symmetry code: (i) 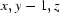.

**Table 2:** Hydrogen-bond geometry (Å, °)

*D*—H⋯*A*	*D*—H	H⋯*A*	*D*⋯*A*	*D*—H⋯*A*
C3—H3*B*⋯O3^ii^	0.97	2.47	3.357 (6)	153
C7—H7⋯O3^iii^	0.93	2.51	3.172 (6)	128
C8—H8⋯O4^i^	0.93	2.47	3.010 (6)	117
C10—H10*A*⋯O2^iv^	0.97	2.50	3.423 (7)	159
C14—H14⋯O1^v^	0.93	2.51	3.419 (7)	167

Symmetry codes: (i) 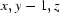; (ii) 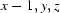; (iii) 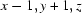; (iv) 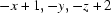; (v) 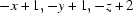.

**Fig. 1.**
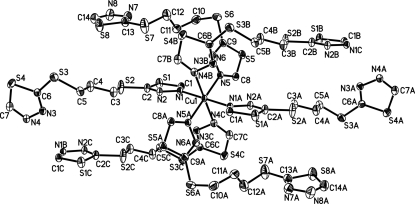
A view of the local coordination of the Cu(II) cation in the title compound. Displacement ellipsoids are drawn at the 30% probability level. The H atoms and perchlorate ion were omitted for clarity. Symmetry codes: (A) -x + 1, -y, -z + 1; (B) x, y - 1, z; (C) -x + 1, -y + 1, -z + 1.

**Fig. 2.**
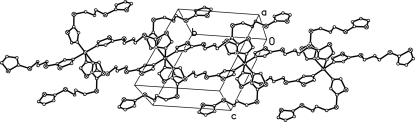
A view of the polymeric chain in the title compound.

**Fig. 3.**
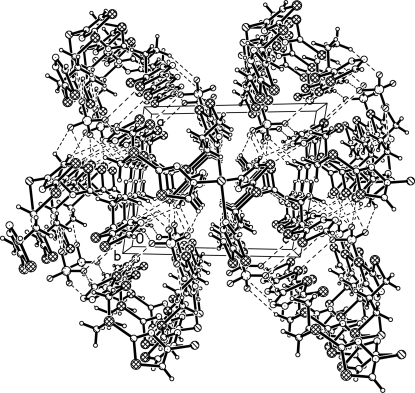
A view of the compound packing down the b axis.

## supplementary crystallographic information

### Comment

The asymmetric structure unit of the title compound consists of a half Cu(II) atom, two [1,3-propanediylbis(thio)]bis[1,3,4-thiadiazole] ligands L, and one perchlorate ion. As depicted in Fig. 1,the Cu(II) atom is coordinated by six N atoms from six ligands L in a slightly distorted octahedral geometry of the central atom. All six Cu—N bond distances are within the range expected for such coordination bonds (Tab. 1) (Huang *et al.*, 2009; Wang *et al.,* 2008). The ligand L adopts two kinds of coordination modes in the crystal structure. One *N,N*-bidentate bridging mode in trans configuration for bridging the copper atom into a one-dimensional chain, with the bridged Cu-Cu distance of 11.122 (3) Å (Fig. 2). The centroid separation and dihedral angle of thiadiazole rings are 9.131 (2) Å and 74.09 (8) °, respectively. The other thiadiazole ligands adopt monodentate coordination mode and serve to complete the octahedral coordination sphere of the copper atom. The corresponding centroid separation and dihedral angle are 8.1499 (16) Å and 65.04 (12) °, respectively. The region between the chains is taken up by uncoordinated perchlorate ions. The perchlorate ions serve as acceptor for C—H···O hydrogen-bonds, which link the chains into a three-dimensional network (Tab. 2. & Fig. 3).

### Experimental

The reaction of [1,3-propanediylbis(thio)]bis[1,3,4-thiadiazole] (0.4 mmol) with Cu(ClO_4_)_2_ (0.1 mmol) in MeOH(10 ml) for a few minutes afforded a light blue solid, which was filtered, washed with acetone, and dried on air. The single crystals suitable for X-ray analysis were obtained by slow diffusion of Et_2_O into the acetonitrile solution of the solid.

### Refinement

All hydrogen atoms were positioned geometrically and treated as riding, with C—H = 0.93 Å (CH) and *U*_iso_(H) = 1.2*U*_eq_(C), with C—H = 0.97 Å (CH2) and *U*_iso_(H) = 1.2*U*_eq_(C).

### Crystal data

**Table:**

[Cu(C_7_H_8_N_4_S_4_)_4_](ClO_4_)_2_	*Z* = 1
*M**_r_* = 1368.10	*F*(000) = 695
Triclinic, *P*1	*D*_x_ = 1.731 Mg m^−^^3^
Hall symbol: -P 1	Mo *K*α radiation, λ = 0.71073 Å
*a* = 10.321 (3) Å	Cell parameters from 4352 reflections
*b* = 11.122 (3) Å	θ = 2.5–28.1°
*c* = 12.908 (4) Å	µ = 1.22 mm^−^^1^
α = 67.213 (3)°	*T* = 294 K
β = 76.602 (3)°	Block, blue
γ = 76.675 (3)°	0.39 × 0.28 × 0.24 mm
*V* = 1312.3 (6) Å^3^	

### Data collection

**Table:**

Bruker SMART CCD area-detector diffractometer	4857 independent reflections
Radiation source: fine-focus sealed tube	4081 reflections with *I* > 2σ(*I*)
graphite	*R*_int_ = 0.017
φ and ω scans	θ_max_ = 25.5°, θ_min_ = 2.5°
Absorption correction: multi-scan (*SADABS*; Bruker, 1997)	*h* = −12→12
*T*_min_ = 0.646, *T*_max_ = 0.756	*k* = −13→13
9833 measured reflections	*l* = −15→15

### Refinement

**Table:**

Refinement on *F*^2^	Primary atom site location: structure-invariant direct methods
Least-squares matrix: full	Secondary atom site location: difference Fourier map
*R*[*F*^2^ > 2σ(*F*^2^)] = 0.040	Hydrogen site location: inferred from neighbouring sites
*wR*(*F*^2^) = 0.110	H-atom parameters constrained
*S* = 1.03	*w* = 1/[σ^2^(*F*_o_^2^) + (0.0533*P*)^2^ + 1.507*P*] where *P* = (*F*_o_^2^ + 2*F*_c_^2^)/3
4857 reflections	(Δ/σ)_max_ < 0.001
322 parameters	Δρ_max_ = 1.05 e Å^−^^3^
0 restraints	Δρ_min_ = −0.50 e Å^−^^3^

### Fractional atomic coordinates and isotropic or equivalent isotropic displacement parameters (Å^2^)

**Table:**

	*x*	*y*	*z*	*U*_iso_*/*U*_eq_	
Cu1	0.5000	0.0000	0.5000	0.02910 (14)	
Cl1	0.89564 (9)	0.39343 (8)	0.71273 (7)	0.0469 (2)	
S1	0.60750 (10)	0.13642 (9)	0.74944 (8)	0.0494 (2)	
S2	0.42947 (13)	0.39702 (10)	0.69426 (11)	0.0728 (4)	
S3	−0.00185 (9)	0.78315 (9)	0.62689 (9)	0.0516 (2)	
S4	0.02700 (10)	1.01392 (10)	0.68458 (9)	0.0569 (3)	
S5	0.58105 (10)	−0.40131 (8)	0.77434 (7)	0.0458 (2)	
S6	0.33608 (12)	−0.34525 (10)	0.94441 (8)	0.0615 (3)	
S7	0.15521 (18)	0.11517 (12)	0.90859 (13)	0.0951 (5)	
S8	0.11321 (15)	0.35074 (11)	0.97930 (11)	0.0777 (4)	
O1	0.8989 (5)	0.4074 (5)	0.8153 (4)	0.1235 (17)	
O2	0.8069 (6)	0.3095 (6)	0.7325 (4)	0.156 (2)	
O3	1.0235 (4)	0.3459 (4)	0.6637 (4)	0.1009 (12)	
O4	0.8505 (4)	0.5164 (4)	0.6350 (4)	0.1255 (17)	
N1	0.5187 (2)	0.0858 (2)	0.6070 (2)	0.0307 (5)	
N2	0.4504 (3)	0.2109 (2)	0.6008 (2)	0.0347 (6)	
N3	0.2075 (2)	0.9204 (2)	0.5508 (2)	0.0371 (6)	
N4	0.2550 (3)	1.0248 (2)	0.5561 (2)	0.0376 (6)	
N5	0.5102 (3)	−0.1771 (2)	0.6333 (2)	0.0336 (5)	
N6	0.4134 (3)	−0.1871 (2)	0.7283 (2)	0.0399 (6)	
N7	0.2510 (4)	0.1319 (3)	1.0804 (3)	0.0609 (9)	
N8	0.2522 (4)	0.2173 (4)	1.1350 (3)	0.0696 (10)	
C1	0.6013 (3)	0.0364 (3)	0.6800 (3)	0.0366 (7)	
H1	0.6529	−0.0470	0.6933	0.044*	
C2	0.4879 (3)	0.2496 (3)	0.6710 (3)	0.0402 (7)	
C3	0.3046 (4)	0.4799 (3)	0.6013 (3)	0.0510 (9)	
H3A	0.3478	0.5122	0.5226	0.061*	
H3B	0.2451	0.4200	0.6088	0.061*	
C4	0.2261 (4)	0.5940 (3)	0.6378 (4)	0.0560 (10)	
H4A	0.2884	0.6480	0.6366	0.067*	
H4B	0.1801	0.5594	0.7154	0.067*	
C5	0.1235 (4)	0.6790 (3)	0.5613 (3)	0.0466 (8)	
H5A	0.0789	0.6227	0.5442	0.056*	
H5B	0.1696	0.7341	0.4901	0.056*	
C6	0.0906 (3)	0.9029 (3)	0.6139 (3)	0.0376 (7)	
C7	0.1716 (3)	1.0817 (3)	0.6212 (3)	0.0462 (8)	
H7	0.1887	1.1538	0.6326	0.055*	
C8	0.6026 (3)	−0.2808 (3)	0.6447 (3)	0.0381 (7)	
H8	0.6739	−0.2882	0.5873	0.046*	
C9	0.4384 (3)	−0.3003 (3)	0.8092 (3)	0.0418 (7)	
C10	0.3970 (5)	−0.2527 (4)	1.0086 (3)	0.0651 (11)	
H10A	0.3613	−0.2815	1.0893	0.078*	
H10B	0.4945	−0.2750	1.0011	0.078*	
C11	0.3601 (5)	−0.1024 (4)	0.9580 (3)	0.0645 (11)	
H11A	0.4071	−0.0620	0.9898	0.077*	
H11B	0.3898	−0.0730	0.8764	0.077*	
C12	0.2129 (5)	−0.0586 (4)	0.9817 (4)	0.0760 (13)	
H12A	0.1868	−0.0778	1.0630	0.091*	
H12B	0.1665	−0.1103	0.9608	0.091*	
C13	0.1830 (4)	0.1885 (4)	0.9971 (3)	0.0544 (9)	
C14	0.1852 (4)	0.3311 (4)	1.0921 (4)	0.0614 (10)	
H14	0.1762	0.3984	1.1203	0.074*	

### Atomic displacement parameters (Å^2^)

**Table:**

	*U*^11^	*U*^22^	*U*^33^	*U*^12^	*U*^13^	*U*^23^
Cu1	0.0416 (3)	0.0211 (2)	0.0251 (2)	0.00102 (19)	−0.0102 (2)	−0.00926 (19)
Cl1	0.0481 (5)	0.0429 (4)	0.0516 (5)	−0.0084 (4)	−0.0051 (4)	−0.0196 (4)
S1	0.0611 (6)	0.0441 (5)	0.0574 (5)	0.0031 (4)	−0.0342 (4)	−0.0256 (4)
S2	0.0904 (8)	0.0534 (6)	0.1059 (9)	0.0226 (5)	−0.0577 (7)	−0.0569 (6)
S3	0.0360 (4)	0.0434 (5)	0.0709 (6)	−0.0081 (4)	−0.0010 (4)	−0.0186 (4)
S4	0.0479 (5)	0.0579 (6)	0.0659 (6)	−0.0016 (4)	0.0071 (4)	−0.0358 (5)
S5	0.0629 (5)	0.0276 (4)	0.0415 (4)	0.0035 (4)	−0.0184 (4)	−0.0068 (3)
S6	0.0777 (7)	0.0484 (5)	0.0432 (5)	−0.0163 (5)	0.0037 (5)	−0.0036 (4)
S7	0.1475 (13)	0.0572 (7)	0.0988 (10)	0.0182 (8)	−0.0737 (10)	−0.0357 (7)
S8	0.1132 (10)	0.0470 (6)	0.0778 (8)	0.0187 (6)	−0.0536 (7)	−0.0231 (5)
O1	0.136 (4)	0.153 (4)	0.130 (3)	0.027 (3)	−0.063 (3)	−0.107 (3)
O2	0.191 (5)	0.200 (5)	0.137 (4)	−0.153 (5)	0.053 (4)	−0.098 (4)
O3	0.072 (2)	0.086 (2)	0.125 (3)	0.0190 (18)	0.004 (2)	−0.045 (2)
O4	0.104 (3)	0.091 (3)	0.107 (3)	0.038 (2)	−0.002 (2)	0.005 (2)
N1	0.0351 (13)	0.0263 (12)	0.0310 (12)	0.0002 (10)	−0.0080 (10)	−0.0118 (10)
N2	0.0408 (14)	0.0289 (12)	0.0397 (14)	0.0025 (10)	−0.0136 (11)	−0.0183 (11)
N3	0.0336 (13)	0.0342 (13)	0.0441 (15)	−0.0026 (10)	−0.0057 (11)	−0.0162 (11)
N4	0.0381 (14)	0.0330 (13)	0.0433 (15)	−0.0018 (11)	−0.0092 (11)	−0.0155 (12)
N5	0.0445 (14)	0.0261 (12)	0.0295 (13)	−0.0005 (10)	−0.0091 (11)	−0.0103 (10)
N6	0.0473 (15)	0.0330 (13)	0.0338 (14)	−0.0022 (11)	−0.0054 (11)	−0.0085 (11)
N7	0.071 (2)	0.0506 (18)	0.0563 (19)	0.0094 (16)	−0.0230 (17)	−0.0175 (16)
N8	0.086 (3)	0.064 (2)	0.064 (2)	0.0160 (19)	−0.0347 (19)	−0.0300 (18)
C1	0.0423 (17)	0.0323 (15)	0.0371 (16)	0.0015 (13)	−0.0144 (13)	−0.0140 (13)
C2	0.0447 (18)	0.0357 (16)	0.0486 (18)	0.0006 (14)	−0.0164 (14)	−0.0228 (15)
C3	0.053 (2)	0.0424 (19)	0.064 (2)	0.0020 (16)	−0.0183 (18)	−0.0267 (18)
C4	0.066 (2)	0.0387 (19)	0.070 (3)	0.0057 (17)	−0.024 (2)	−0.0267 (18)
C5	0.051 (2)	0.0367 (17)	0.056 (2)	−0.0069 (15)	−0.0110 (16)	−0.0183 (16)
C6	0.0355 (16)	0.0337 (16)	0.0398 (17)	0.0008 (13)	−0.0064 (13)	−0.0123 (13)
C7	0.050 (2)	0.0391 (18)	0.054 (2)	0.0009 (15)	−0.0135 (16)	−0.0217 (16)
C8	0.0463 (18)	0.0332 (16)	0.0357 (16)	0.0015 (13)	−0.0133 (13)	−0.0135 (13)
C9	0.054 (2)	0.0306 (16)	0.0377 (17)	−0.0075 (14)	−0.0097 (14)	−0.0073 (13)
C10	0.078 (3)	0.064 (3)	0.046 (2)	0.000 (2)	−0.0054 (19)	−0.0201 (19)
C11	0.084 (3)	0.064 (3)	0.049 (2)	−0.021 (2)	−0.001 (2)	−0.024 (2)
C12	0.088 (3)	0.052 (2)	0.087 (3)	0.001 (2)	−0.020 (3)	−0.027 (2)
C13	0.065 (2)	0.0404 (19)	0.053 (2)	0.0016 (17)	−0.0184 (18)	−0.0126 (17)
C14	0.071 (3)	0.054 (2)	0.062 (2)	0.004 (2)	−0.020 (2)	−0.025 (2)

### Geometric parameters (Å, °)

**Table:**

Cu1—N1^i^	2.021 (2)	N3—N4	1.390 (4)
Cu1—N1	2.021 (2)	N4—C7	1.293 (4)
Cu1—N5^i^	2.053 (2)	N4—Cu1^iv^	2.445 (3)
Cu1—N5	2.053 (2)	N5—C8	1.299 (4)
Cu1—N4^ii^	2.445 (3)	N5—N6	1.375 (3)
Cu1—N4^iii^	2.445 (3)	N6—C9	1.304 (4)
Cl1—O2	1.370 (4)	N7—C13	1.291 (5)
Cl1—O4	1.398 (4)	N7—N8	1.387 (5)
Cl1—O3	1.404 (3)	N8—C14	1.271 (5)
Cl1—O1	1.400 (4)	C1—H1	0.9300
S1—C1	1.696 (3)	C3—C4	1.517 (5)
S1—C2	1.735 (3)	C3—H3A	0.9700
S2—C2	1.727 (3)	C3—H3B	0.9700
S2—C3	1.806 (4)	C4—C5	1.514 (5)
S3—C6	1.746 (3)	C4—H4A	0.9700
S3—C5	1.815 (4)	C4—H4B	0.9700
S4—C7	1.714 (4)	C5—H5A	0.9700
S4—C6	1.735 (3)	C5—H5B	0.9700
S5—C8	1.695 (3)	C7—H7	0.9300
S5—C9	1.719 (3)	C8—H8	0.9300
S6—C9	1.767 (3)	C10—C11	1.530 (6)
S6—C10	1.829 (5)	C10—H10A	0.9700
S7—C13	1.742 (4)	C10—H10B	0.9700
S7—C12	1.816 (5)	C11—C12	1.483 (6)
S8—C14	1.703 (4)	C11—H11A	0.9700
S8—C13	1.727 (4)	C11—H11B	0.9700
N1—C1	1.293 (4)	C12—H12A	0.9700
N1—N2	1.388 (3)	C12—H12B	0.9700
N2—C2	1.302 (4)	C14—H14	0.9300
N3—C6	1.299 (4)		
			
N1^i^—Cu1—N1	180.0	C4—C3—H3B	110.5
N1^i^—Cu1—N5^i^	88.01 (9)	S2—C3—H3B	110.5
N1—Cu1—N5^i^	91.99 (9)	H3A—C3—H3B	108.7
N1^i^—Cu1—N5	91.99 (9)	C3—C4—C5	112.1 (3)
N1—Cu1—N5	88.01 (9)	C3—C4—H4A	109.2
N5^i^—Cu1—N5	180.0	C5—C4—H4A	109.2
N1^i^—Cu1—N4^ii^	91.50 (9)	C3—C4—H4B	109.2
N1—Cu1—N4^ii^	88.50 (9)	C5—C4—H4B	109.2
N5^i^—Cu1—N4^ii^	87.34 (9)	H4A—C4—H4B	107.9
N5—Cu1—N4^ii^	92.66 (9)	C4—C5—S3	111.8 (3)
N1^i^—Cu1—N4^iii^	88.50 (9)	C4—C5—H5A	109.3
N1—Cu1—N4^iii^	91.50 (9)	S3—C5—H5A	109.3
N5^i^—Cu1—N4^iii^	92.66 (9)	C4—C5—H5B	109.3
N5—Cu1—N4^iii^	87.34 (9)	S3—C5—H5B	109.3
N4^ii^—Cu1—N4^iii^	180.0	H5A—C5—H5B	107.9
O2—Cl1—O4	108.6 (4)	N3—C6—S4	114.1 (2)
O2—Cl1—O3	110.1 (3)	N3—C6—S3	125.0 (2)
O4—Cl1—O3	107.8 (2)	S4—C6—S3	120.83 (18)
O2—Cl1—O1	108.9 (3)	N4—C7—S4	114.8 (3)
O4—Cl1—O1	109.3 (3)	N4—C7—H7	122.6
O3—Cl1—O1	112.2 (3)	S4—C7—H7	122.6
C1—S1—C2	86.96 (14)	N5—C8—S5	113.6 (2)
C2—S2—C3	103.64 (16)	N5—C8—H8	123.2
C6—S3—C5	101.32 (16)	S5—C8—H8	123.2
C7—S4—C6	86.73 (16)	N6—C9—S5	114.4 (2)
C8—S5—C9	87.49 (15)	N6—C9—S6	122.9 (3)
C9—S6—C10	99.43 (18)	S5—C9—S6	122.70 (18)
C13—S7—C12	102.4 (2)	C11—C10—S6	115.4 (3)
C14—S8—C13	86.78 (19)	C11—C10—H10A	108.4
C1—N1—N2	113.7 (2)	S6—C10—H10A	108.4
C1—N1—Cu1	124.3 (2)	C11—C10—H10B	108.4
N2—N1—Cu1	121.73 (17)	S6—C10—H10B	108.4
C2—N2—N1	110.3 (2)	H10A—C10—H10B	107.5
C6—N3—N4	111.9 (2)	C12—C11—C10	111.8 (4)
C7—N4—N3	112.5 (3)	C12—C11—H11A	109.2
C7—N4—Cu1^iv^	133.3 (2)	C10—C11—H11A	109.2
N3—N4—Cu1^iv^	109.59 (17)	C12—C11—H11B	109.2
C8—N5—N6	113.8 (2)	C10—C11—H11B	109.2
C8—N5—Cu1	128.7 (2)	H11A—C11—H11B	107.9
N6—N5—Cu1	117.31 (18)	C11—C12—S7	115.4 (4)
C9—N6—N5	110.7 (3)	C11—C12—H12A	108.4
C13—N7—N8	111.7 (3)	S7—C12—H12A	108.4
C14—N8—N7	112.8 (3)	C11—C12—H12B	108.4
N1—C1—S1	114.4 (2)	S7—C12—H12B	108.4
N1—C1—H1	122.8	H12A—C12—H12B	107.5
S1—C1—H1	122.8	N7—C13—S8	113.8 (3)
N2—C2—S2	127.1 (2)	N7—C13—S7	126.1 (3)
N2—C2—S1	114.6 (2)	S8—C13—S7	120.0 (2)
S2—C2—S1	118.30 (18)	N8—C14—S8	114.9 (3)
C4—C3—S2	106.2 (2)	N8—C14—H14	122.5
C4—C3—H3A	110.5	S8—C14—H14	122.5
S2—C3—H3A	110.5		
			
N5^i^—Cu1—N1—C1	−137.6 (3)	S2—C3—C4—C5	175.9 (3)
N5—Cu1—N1—C1	42.4 (3)	C3—C4—C5—S3	163.0 (3)
N4^ii^—Cu1—N1—C1	−50.3 (3)	C6—S3—C5—C4	74.5 (3)
N4^iii^—Cu1—N1—C1	129.7 (3)	N4—N3—C6—S4	0.6 (3)
N5^i^—Cu1—N1—N2	36.6 (2)	N4—N3—C6—S3	178.9 (2)
N5—Cu1—N1—N2	−143.4 (2)	C7—S4—C6—N3	−0.2 (3)
N4^ii^—Cu1—N1—N2	123.9 (2)	C7—S4—C6—S3	−178.5 (2)
N4^iii^—Cu1—N1—N2	−56.1 (2)	C5—S3—C6—N3	12.7 (3)
C1—N1—N2—C2	0.6 (4)	C5—S3—C6—S4	−169.1 (2)
Cu1—N1—N2—C2	−174.2 (2)	N3—N4—C7—S4	0.7 (4)
C6—N3—N4—C7	−0.8 (4)	Cu1^iv^—N4—C7—S4	−151.87 (18)
C6—N3—N4—Cu1^iv^	158.3 (2)	C6—S4—C7—N4	−0.3 (3)
N1^i^—Cu1—N5—C8	65.1 (3)	N6—N5—C8—S5	−0.5 (3)
N1—Cu1—N5—C8	−114.9 (3)	Cu1—N5—C8—S5	173.93 (14)
N4^ii^—Cu1—N5—C8	−26.5 (3)	C9—S5—C8—N5	0.4 (3)
N4^iii^—Cu1—N5—C8	153.5 (3)	N5—N6—C9—S5	−0.1 (3)
N1^i^—Cu1—N5—N6	−120.6 (2)	N5—N6—C9—S6	178.9 (2)
N1—Cu1—N5—N6	59.4 (2)	C8—S5—C9—N6	−0.1 (3)
N4^ii^—Cu1—N5—N6	147.8 (2)	C8—S5—C9—S6	−179.1 (2)
N4^iii^—Cu1—N5—N6	−32.2 (2)	C10—S6—C9—N6	−78.7 (3)
C8—N5—N6—C9	0.4 (4)	C10—S6—C9—S5	100.2 (2)
Cu1—N5—N6—C9	−174.7 (2)	C9—S6—C10—C11	69.0 (3)
C13—N7—N8—C14	0.8 (6)	S6—C10—C11—C12	66.9 (4)
N2—N1—C1—S1	−0.6 (3)	C10—C11—C12—S7	−171.5 (3)
Cu1—N1—C1—S1	174.06 (14)	C13—S7—C12—C11	−83.7 (4)
C2—S1—C1—N1	0.3 (3)	N8—N7—C13—S8	−0.6 (5)
N1—N2—C2—S2	−179.2 (2)	N8—N7—C13—S7	−179.1 (3)
N1—N2—C2—S1	−0.4 (3)	C14—S8—C13—N7	0.2 (4)
C3—S2—C2—N2	1.0 (4)	C14—S8—C13—S7	178.8 (3)
C3—S2—C2—S1	−177.8 (2)	C12—S7—C13—N7	11.3 (5)
C1—S1—C2—N2	0.1 (3)	C12—S7—C13—S8	−167.1 (3)
C1—S1—C2—S2	179.0 (2)	N7—N8—C14—S8	−0.6 (5)
C2—S2—C3—C4	166.7 (3)	C13—S8—C14—N8	0.2 (4)

Symmetry codes: (i) −*x*+1, −*y*, −*z*+1; (ii) −*x*+1, −*y*+1, −*z*+1; (iii) *x*, *y*−1, *z*; (iv) *x*, *y*+1, *z*.

### Hydrogen-bond geometry (Å, °)

**Table:**

*D*—H···*A*	*D*—H	H···*A*	*D*···*A*	*D*—H···*A*
C3—H3B···O3^v^	0.97	2.47	3.357 (6)	153
C7—H7···O3^vi^	0.93	2.51	3.172 (6)	128
C8—H8···O4^iii^	0.93	2.47	3.010 (6)	117
C10—H10A···O2^vii^	0.97	2.50	3.423 (7)	159
C14—H14···O1^viii^	0.93	2.51	3.419 (7)	167

Symmetry codes: (v) *x*−1, *y*, *z*; (vi) *x*−1, *y*+1, *z*; (iii) *x*, *y*−1, *z*; (vii) −*x*+1, −*y*, −*z*+2; (viii) −*x*+1, −*y*+1, −*z*+2.
